# Effectiveness of Leaf Rust Resistance Genes in the Adult and Juvenile Stages in Southern Russia in 2011–2020

**DOI:** 10.3390/plants11060793

**Published:** 2022-03-16

**Authors:** Galina Volkova, Olga Kudinova, Olga Vaganova, Valeria Agapova

**Affiliations:** Ministry of Science and Higher Education of the Russian Federation, Federal Scientific Center for Biological Plant Protection, 350039 Krasnodar, Russia; galvol@bpp.yandex.ru (G.V.); vof54@mail.ru (O.V.); agapovalera1996@gmail.com (V.A.)

**Keywords:** leaf rust, *Puccinia triticina*, resistance genes, APR-genes, ASR-genes, population, effectiveness

## Abstract

*Puccinia triticina* Erikss. is a causative agent of wheat leaf rust spread worldwide. Wheat rust is a major disease on wheat in southern regions of Russia, which are leaders in grain production and have favorable conditions for pathogen development. In this paper we studied the effectiveness of 52 NILs of cv. Thatcher with *Lr* genes in field trials and 41 NILs—in the juvenile phase in a greenhouse during 2011–2020. We conclude that the lines with *Lr9*, *Lr42* and *Lr43+24* genes remained immune in the adult phase during ten years of research. Lines with *Lr* genes: *19*, *24*, *29*, *36*, *37*, *38*, *43*, *45*, *47*, *50* showed efficiency in field tests (1–5 R on the CIMMYT scale). No immune lines to *Puccinia triticina* were registered in the juvenile phase during 2011–2020. The line with the *Lr9* gene remained immune up to 2020; *Lr19* and *Lr41*—up to 2015; *Lr42*—up to 2018, and *Lr50*—up to 2019. In 2020, there was an increase of *P. triticina* isolates with virulence to Thatcher lines with *Lr*: *9*, *14a*, *16*, *19*, *21*, *28*, *30*, *33*, *40*, *45*, *W*, *50*. Additionally, we registered a change in infection types towards more susceptible in isogenic *Lr* gene lines: *1*, *2a*, *12*, *14b*, *15*, *18*, *20*, *23*, *25*, *28*, *29*, *32*, *35*, *36*, *37*, *38*, *40*, *44*, *45* in the field. A sharp increase in the frequencies of virulent isolates was recorded in 2018–2020 due to unfavorable weather in the growing seasons. This indicates the ability of a dangerous pathogen to rapidly evolve in response to biotic and abiotic stresses. Therefore, annual monitoring of the reaction of isogenic lines, selected released varieties and the study of the virulence of the phytopathogen are important measures necessary to prevent and control leaf rust in grain-producing regions of the world.

## 1. Introduction

*Puccinia triticina* Erikss. is a causative agent of wheat leaf rust. It is an obligate biotrophic parasite spread worldwide. Statistics indicate that Russia is among the top five in world wheat production [1]. According to the Expert Analytical Center for Agribusiness, in 2020 the area under wheat in Russia amounted to 29.4 thousand hectares, which is 36.9% of the total area of all crops (https://ab-centre.ru/news/posevnye-ploschadi-i-sbory-osnovnyh-selskohozyaystvennyh-kultur-itogi-za2020-god (accessed on 16 July 2021)).

The main grain-producing geographical regions are located in Southern Russia: Rostov Region, Krasnodar region and Stavropol region. Their total gross harvest of grain is 35.4% of the total harvest in the country. Leaf rust is a major disease on wheat inSouthern Russia. From 1971 to 1998, leaf rust epiphytoties were periodically monitored in the region. This is due to an increase in the sown area under the leader varieties and overcoming the resistance of these varieties by the virulent pathotypes of *P. triticina*. The frequency of occurrence of leaf rust epidemics has reduced drastically from 1998 to the present. This is due to an increase in the genetic diversity of sown varieties and the absence of a leader variety [2]. However, despite the advances in modern breeding of rust-resistant wheat varieties, leaf rust is still a harmful disease that must be continuously monitored. To do so, it is necessary to track annual changes in the virulence of the pathogen population and evaluate the effectiveness of the known leaf rust resistance genes. In Russia, such studies are carried out in the main scientific centers located in different geographical regions. In the Northwest, it is All-Russian Institute of Plant Protection [3]; in the central part—All-Russian Institute of Phytopathology [4]; in the Volga region—Federal Center of Agriculture Research of the South—East Region [5]; in the south—Federal Scientific Center for Biological Plant Protection [6]; and in Siberia—Institute of Cytology and Genetics [7].

To date, about 80 leaf rust resistant genes are known [8]. Most of them are race specific resistance or all stage resistance (ASR) genes. Such resistance is not “durable” because it’s controlled by one or more genes [9,10]. The evolving nature of plant pathogens results in new virulent races that rapidly overcome ASR genes [11,12]. Race non-specific resistance genes (APR genes)—*Lr34*, *Lr46*, *Lr67* and *Lr68*—are usually susceptible at the stage of seedlings. But at the stage of adult plants they exhibit slow rusting, prolonging plant resistance [13,14]. Combinations of several APR and ASR genes are especially effective [15,16]. Therefore, it is necessary to establish the effectiveness of *Lr* resistance genes in different phases of plant development in retrospect for successful wheat breeding programs.

The aim of this paper was to evaluate the effectiveness of *Lr* genes in the adult and juvenile phases of plants in Southern Russia in 2011–2020.

## 2. Results

### 2.1. Efficiency of Lr Genes in Adult Phase

Table 1 presents the evaluation results of a set of near isogenic lines of wheat cv. Thatcher. Lines with genes *Lr9*, *Lr43* and *Lr50* show absolute efficiency (no signs of disease on plants) in the field tests in 2011–2020. Lines with *Lr42* and *Lr43+24* genes had no signs of leaf rust in 2011–2019;but in 2020 they had minimal infection by infection type R (according to the CIMMYT scale) [17]. Effective (1R–5R) *Lr* genes are the following: *19*, *24*, *29*, *36*, *37*, *38*, *45*, *47*; moderately effective (10MR–20MR) *Lr* genes: *17*, *21*, *22a*, *41*, *52*. Thatcher lines with known *Lr* resistance genes: *1*, *2a*, *2b*, *2c*, *3*, *3bg*, *3ka*, *10*, *11*, *13*, *14a*, *14b*, *15*, *16*, *20*, *22b*, *23*, *26*, *30*, *33*, *34*, *40*, *B*, *Kanred* were ineffective in the adult phase. Their prevalence varied within 20MS–90S (Table 1). Lines with *Lr* genes: *12*, *18*, *25*, *28*, *32*, *35*, *36*, *37* were moderately effective from 2011 to 2019, but in 2020 they showed susceptibility to *P. triticina*.

### 2.2. Efficiency of Lr Genes in Juvenile Phase

We assessed the resistance of Thatcher isogenic lines to *P. triticina* isolates in the greenhouse conditions of the Federal Scientific Center for Biological Plant Protection in 2011–2020. The results are described in Table 2. All lines with *Lr* genes were susceptible to *P. triticina* infection during the study period. Previously effective *Lr9* became susceptible to virulent isolates of the fungus in 2020 (with a frequency of 12.8%). Isolates virulent to *Lr24* were encountered in a small number years earlier [18], but in 2018 and 2020 their number increased to 20.0% and 14.8%, respectively.

The percentage of virulence frequency of *P. triticina* isolates to lines with the following *Lr* genes: *2c*, *3*, *3ka*, *11*, *14b*, *23*, *26*, *28*, *33*, *40*, *B*, *Exch*, *Kanred* was high (25–90%) during the entire study period. The frequency of P. triticina isolates virulence to Thatcher lines with *Lr*: *2a*, *3bg*, *10*, *14a*, *16*, *17*, *20*, *21*, *25*, *30*, *32* varied over the research years. The frequency of virulent isolates to *Lr36*, *Lr38* increased significantly in 2018–2020. *P. triticina* isolates with virulence to *Lr: 1*, *18*, *19*, *44*, *45*, *W* increased during the study period. Isolates with virulence to *Lr:15*, *29*, *42*, *43+24* were rare (0 to 25%). At the same time, the line with the *Lr42* gene was not affected by leaf rust from 2011 to 2017, and in 2018–2020, the frequency of virulence isolates was 1.7–7.5%. An increase in frequency *P. triticina* isolates with virulence to *Lr 43+24* was observed from 2017 to 2019 (4.7–9.1%). The line with *Lr47* was not affected until 2018, and the percentage of virulent isolates to *Lr41* was low (2.2–8.8%), except for 2015.

## 3. Discussion

A ten-year study of the *Lr* genes effectiveness in different stages of plant development revealed that *Lr9*, *Lr42*, and *Lr43* are immune to wheat rust both in seedling and adult phase in Southern Russia. At the same time, on the lines with genes *Lr42*, *Lr43+24*, disease symptoms first appeared in 2020 in adult phase. In a number of regions of Russia (Urals, Western Siberia), *Lr9* (transferred to soft wheat from *Aegilops umbellulata*) has lost its effectiveness since 2007 due to the spread of varieties containing this gene [19,20]. In Moscow region, isolates virulent to *Lr9* were encountered with a low frequency over the period from 2009 to 2017, but in 2013 their share was already 75% [21]. At the same time, in the Volga region, as in Dagestan, *Lr9* is still effective [5,22]. *Lr 42*, which was transferred to soft wheat from *Aegilops tauschii* Coss [23], maintains absolute efficiency in all major grain-sowing regions of Russia [4]. But in Ukraine, *Lr42* is effective only in field trials; and in the seedling stage, this line shows moderate susceptibility [24].

In 2004–2006, the list of ASR genes studied in Southern Russia was longer and included the following genes transferred to common wheat from wild forms: *Lr9*, *Lr19*, *Lr24*, *Lr29*, *Lr38*, *Lr41*, *Lr42*, *Lr43*, *Lr45* [25]. Most of these *Lr* genes have now lost their effectiveness in the juvenile phase.

**Figure 1:**
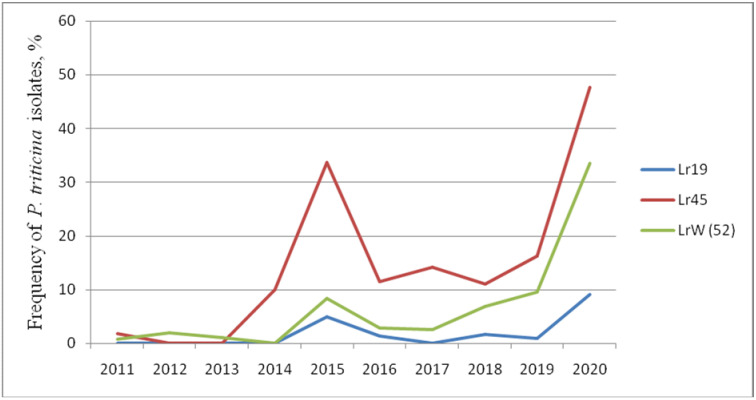
Efficiency dynamics of *Lr* genes in the seedling phase against the North Caucasian population of *P. triticina* (2011–2020).

Thus, genes *Lr: 19*, *24*, *29*, *36*, *37*, *38*, *45*, *47*, *17*, *21*, *22a*, *41*, *W (52)* that are effective and moderately effective under field trials show different reactions to infection with *P. triticina* isolates in the seedling phase. The frequency of virulent isolates to *Lr19*, *Lr45*, and *W (52)* gradually increased, reaching its peak in 2020 (Figure 1). The *Lr17*, *Lr21*, *Lr36*, and *Lr38* genes were ineffective in the juvenile phase. The frequency of isolates with virulence to these lines varied from 0 to 96.5% in different years. *Lr24* decreased its effectiveness over time, and moderate virulence to this line (up to 20%) was observed in 2018 and 2020. The line with the *Lr29* gene was affected by fungus isolates in different years from 0 to 21.4%.

*Lr19* and *Lr24*, introgressed into soft wheat from *Aegilops elongatum* [26], have a different history of their efficiency in Russia. *Lr19*, due to its active use in the composition of varieties and lines in breeding, lost its effectiveness in the juvenile phase in a number of regions of Russia: the Volga region [22], Western Siberia [27], and the Central part of Russia [21]. The effectiveness of *Lr19* still contributes to wheat breeding in the Southern Urals [20], as well as in Ukraine [24] and Dagestan [22]. To prolong the genetic resistance of *Lr19*, breeders use its combination with various genes: *Lr10*, *Lr26*, *Lr37*, *Lr39* [20]. The *Lr24* gene remains effective in most regions of Russia in the adult phase [27] and in the seedling phase [4]. At the same time, in Saratov region, as well as in Ukraine, *Lr24* is no longer effective in the germination phase [5,24]. The appearance and increase in the frequency of isolates virulent to *Lr24* in Southern Russia may indicate a possible introduction of infection from Ukraine, since *Lr24* is scarcely used in Russian breeding [20].

The efficiency of this and other *Lr* genes varies worldwide. For example, in South Africa, effective genes include *Lr9*, *Lr19*, *Lr29*, *Lr34*, *Lr45*, *Lr47*, *Lr51* and *Lr52*. At the same time, varieties and lines containing the race-specific genes *Lr1*, *Lr3a*, *Lr10*, *Lr13*, *Lr14a*, *Lr17b*, *Lr24*, *Lr26*, *Lr27*, and *Lr31* are no longer used, since they were overcome by new races of the pathogen [28]. In Egypt, *Lr9* and *Lr24* are ineffective [29], while molecular markers prove the presence of *Lr13*, *Lr19*, *Lr24*, *Lr26*, *Lr34*, *Lr35 Lr36*, *Lr37*, *Lr39*, and *Lr46* genes in Egyptian varieties [30]. *Lr24* is not effective in Iran either [31]. In Australia, *Lr24* was effective in preventing wheat rust disease from 1983 until 2000, when a variety with this gene was first sown, and when a virulent isolate was found [32]. Australian commercial cultivars contain the following resistance genes: *Lr1*, *Lr3a*, *Lr13*, *Lr13+*, *Lr14a*, *Lr17a*, *Lr17b*, *Lr20*, *Lr23*, *Lr24*, *Lr26*, *Lr27*, *Lr31*, *Lr34*, *Lr37* and *Lr46*. A significant number of cultivars possess *Lr34* as well [33]. Recently, pathotypes virulent to *Lr24* have also been found in New Zealand [12]. In Israel, *Lr24* still retains its effectiveness [34].

Our results suggest the need for both monitoring the virulence of the leaf rust pathogen population and studying the effectiveness of known resistance genes in different phases of plant development. This will help to find new diverse effective resistance genes that can be used for wheat breeding in Russia and worldwide.

## 4. Conclusions

We studied the effectiveness of *Lr* genes against the wheat leaf rust pathogen population in both juvenile and adult phases during 2011–2020 in Southern Russia. We found that the lines with *Lr9*, *Lr42*, and *Lr43+24* genes remained immune throughout ten years of research in all phases of plant development. Lines with *Lr*: *19*, *24*, *29*, *36*, *37*, *38*, *43*, *45*, *47*, *50* showed efficiency in field trials (1R–5R on the CIMMYT scale). In the juvenile phase, no immune lines to *Puccinia triticina* were found in 2011–2020. The line with *Lr9* gene was highly resistant by 2020; *Lr19* and *Lr41*—by 2015; *Lr42*—by 2018; and *Lr50*—by 2019.

Nevertheless, the *Lr9*, *Lr42*, *Lr43+24* genes are able to resist leaf rust in juvenile and adult phases. In 2020, we noted both an increase in the frequencies of virulent isolates to lines with *Lr*: *9*, *14a*, *16*, *19*, *21*, *28*, *30*, *33*, *40*, *45*, *W*, *50*, as well as changes in infection types towards more susceptible in Thatcher lines with *Lr*: *1*, *2a*, *12*, *14b*, *15*, *18*, *20*, *23*, *25*, *28*, *29*, *32*, *35*, *36*, *37*, *38*, *40*, *44*, *45* in field trials. In addition, sharp increases in the frequencies of virulent isolates for a number of *Lr*-lines were noted in 2013, 2018, and 2020. We hypothesize that this is due to the unfavorable weather (relatively low rainfall) in the growing seasons during these years (Figure 1).

Several literature sources mention that virulent isolates may predominate in unfavorable weather [35]. This indicates the ability of a dangerous pathogen to rapidly evolve in response to biotic and abiotic stresses. Of no small importance are the genotypes of varieties grown in the region, which contribute to the selection of virulent isolates of the fungus. The loss of efficiency of the *Lr* genes in the field trials and juvenile phase dictates the need for a more thorough study of resistance genetics of widely planted varieties, including the application of molecular markers. This should be considered for wheat variety selection and placement.

There are few data on the genetics of resistance of breeding varieties in the south of Russia, and such work is our task for the future. Annual monitoring of the effectiveness of isogenic lines, selected released varieties and the study of the pathogen virulence are important measures necessary to prevent and control leaf rust in grain-producing regions of the world.

## 5. Materials and Methods

### 5.1. Growing Seasons 2011–2020

**Figure 2:**
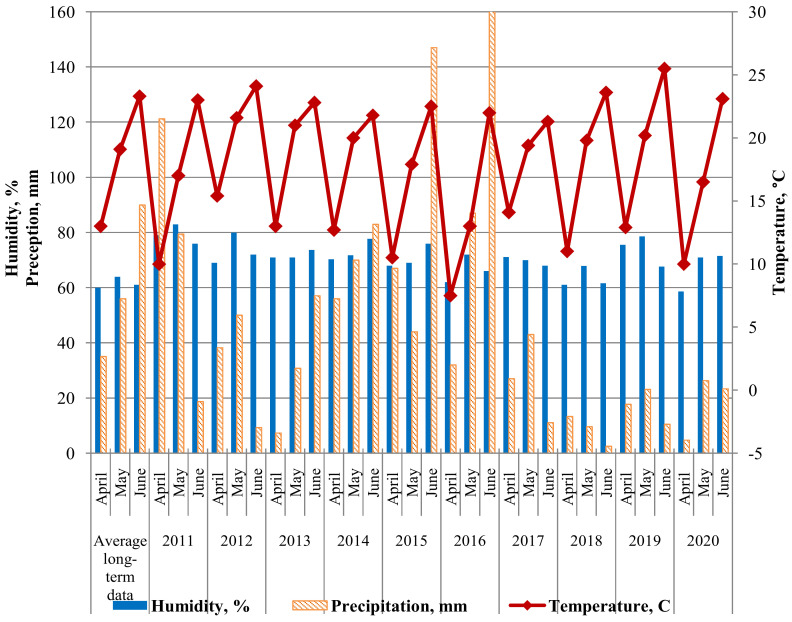
Climatogram of weather conditions for the research period 2011–2020 (according to the FSCBPP meteorological station).

Figure 2 shows the weather of the three most significant months for disease development (April, May, June) during 2011–2020. The data is provided by a weather station at the Federal Scientific Center for Biological Plant Protection, Krasnodar (FSCBPP). The combination of sufficient rainfall and elevated temperatures is typical for most spring months in Southern Russia. This is ideal for leaf rust development [36]. Therefore, most growing seasons were favorable for disease development. The growing seasons of 2012, 2018 and 2020 were an exception, for which a reduced amount of precipitation was noted. In 2012, the lack of precipitation was in June, and in 2020, in April. Lack of precipitation from April to June in 2018 did not contribute to disease development.

### 5.2. Obtaining Infectious Material of P. triticina

We received *P. triticina* infectious material as a result of annual route inspections of industrial and selection crops of winter wheat in Krasnodar region, Stavropol region and Rostov Region during 2011–2020. Affected leaves were collected from each area, signed and placed in a cold storage in filter paper at 4–6 °C. Then leaf samples were mixed to obtain a population of *P. triticina.* For further experiments, the pathogen population was propagated on a susceptible cultivar Michigan Amber.

### 5.3. Efficiency Evaluation of the Lr Genes at the Adult Stage

We carried out the studies at the FSCBPP field site in 2010–2020 using a set of 52 NILs of winter wheat cv. Thatcher containing the following resistance *Lr* genes: *1*, *2a*, *2b*, *2c*, *3*, *3bg*, *3ka*, *9*, *10*, *11*, *12*, *13*, *14a*, *14b*, *15*, *16*, *17*, *18*, *19*, *20*, *21*, *22a*, *22b*, *23*, *24*, *25*, *26*, *28*, *29*, *30*, *32*, *33*, *34*, *35*, *36*, *37*, *38*, *40*, *41*, *42*, *43*, *43+24*, *44*, *45*, *47*, *B*, *50*, *52 (W)*, *57 (58)*, *Exch*, *KR1*, *KR2*, *73.*

Seeds of each line were grown at the FSCBPP field site in autumn on plots of 1 sq.m. Every 10 plots, a susceptible variety was grown, which served as a reservoir of infection. Michigan Amber was the control for susceptibility. Plants were inoculated in the booting phase (Z 32). For plant inoculation, we used a mixture of *P. triticina* urediniospores with talc in a ratio of 1:100 at a load of 5 mg spores/m^2^ [18]. We scored the plants for infection at the time of the initial manifestation of the disease, and then every seven days until the peak incidence.

The severity of damage (in %) and the reaction of wheat to infection (in points) were assessed by the CIMMYT scale [37]. The following scale was used: 0—no spots or uredinia; R—small uredinia with necrosis; MR—moderate size pustules with necrosis; MS-moderate pustules with chlorosis; S—large pustules. The genes were ranked as follows: highly effective (plants without signs of damage), effective (1-5 R), moderately effective (10–20 MR), ineffective (25 MS and above). Ranking was performed according to the CIMMYT scale [17].

### 5.4. Efficiency Evaluation of the Lr Genes at the Juvenile Stage

We carried out the studies in greenhouses of FSCBPP in 2011–2020 using a set of 41 NILs of winter wheat cv. Thatcher containing the following resistance *Lr* genes: *1*, *2a*, *2c*, *3*, *3bg*, *3ka*, *9*, *10*, *11*, *14a*, *14b*, *15*, *16*, *17*, *18*, *19*, *20*, *21*, *23*, *24*, *25*, *26*, *28*, *29*, *30*, *32*, *33*, *36*, *38*, *40*, *41*, *42*, *43+24*, *44*, *45*, *47*, *B*, *52 (W)*, *Exch*, *KR1KR2*, *50.*

The seeds of each line were pre-germinated in Petri dishes, and then sown in pots, 5 plants of each line. Seedlings were grown for 7 days at mean temperature approximately 18- 20 C in isolated greenhouse boxes. Then sets of seedlings of isogenic lines were inoculated with each of the single pustule isolates of the *P. triticina* population. We described in detail the methods of isolation and reproduction of single pustule isolates of the leaf rust pathogen population in the paper [6]. At 14 days after inoculation, plant damage was scored for infection type (IT): low IT 0–2+ and high IT (3,4) according to Kolmer [36,38]. The effectiveness of the Lr genes in the juvenile stage was assessed by the frequency of virulent *P. triticina* isolates to the lines carrying the *Lr* genes.

For our study we used the material and technical base of the unique scientific installation “Phytotron for the isolation, identification, study and maintenance of races, strains, phenotypes of pathogens” (http://ckp-rf.ru/671925 (accessed on 2 February 2021)).

## Figures and Tables

**Table 1:** Immunological assessment of near isogenic Thatcher lines for resistance to the North Caucasian *P. triticina* population (infectious site of FSCBPP, 2011–2020).

Genes Lr	Line Names, RL	Plant Infection Types by Years									
		2011	2012	2013	2014	2015	2016	2017	2018	2019	2020
1	Tc^6^/Centenario RL 6003	70MS	40MS	60MS	80MS	70MS	80MS	80MS	60MS	50MS	60S
2a	Tc^6^/Webster RL 6016	40MS	20MS	50MS	50MS	50MS	50MS	50MS	50MS	30MR	20S
2b	Tc^6^/Carina RL 6019	70S	80S	90S	90S	70S	60S	70S	70S	60S	30S
2c	Tc^6^/Loros RL 6025	60S	80S	90S	100S	90S	80S	80S	80S	70S	55S
3	Tc^6^/Democrat RL 6002	80S	90S	90S	90S	80S	90S	80S	90S	80S	45S
3bg	Bage/Tc^8^ RL 6042	80S	70S	80S	90S	70S	50S	50S	30S	30MS	25S
3ka	Tc^6^/Aniversario RL 6007	80S	60S	90S	90S	70MS	70MS	80MS	80MS	40MS	35S
9	Transfer/Tc^6^ RL 6010	R	0	0	0	0	0	0	0	0	0
10	Tc^6^/Exchange RL 6004	90S	90S	100S	90S	90S	90S	80S	90S	70S	70S
11	Tc^6^/Hussar RL 6053	70S	50S	80S	90S	90S	80S	80S	80S	50S	60S
12	Exchange/Tc^6^ RL 6011	5R	10MR	20MR	30MR	20MR	15MR	15MR	10MR	10R	25S
13	Tc^7^/Frontana RL 4031	80S	60S	70S	90S	80S	80S	70MS	80MS	60S	60S
14a	Selkirk/Tc^6^	70S	60S	80S	80S	30MS	40S	50S	50S	30MR	65S
14b	Tc^6^/M.Escobar RL 6006	70S	40MS	50MS	80MS	70MS	60MS	60MS	70MS	50MS	45S
15	Tc^6^/Kenya W1483 RL 6052	50MS	30MR	40MS	40MS	20MS	20MS	30MS	40MS	30MR	25S
16	Tc^6^/Exchange RL 6005	60S	40S	50S	70MS	50MS	60MS	60MS	60MS	40MS	65S
17	K. Lucero/Tc^6^ RL 6008	7MR	7MR	10MR	15MR	10MR	10MR	10MR	10MR	10R	10MR
18	Tc^7^/Africa 43 RL 6090	5R	5R	15MR	15MR	10MR	10MR	10MR	5MR	5R	15MS
19	Tc^7^/Tr.4A.elong. RL 6040	5R	R	5R	R	R	R	R	R	1R	5R
20	Tc^6^/Timmo RL6092	50MR	20MR	30MS	40MS	60MS	30MS	30MS	40MS	30MR	25S
21	Tc^6^/RL5406 Tetra RL6043	20MR	20MR	40S	40MS	20MR	15MR	15MR	10MR	10R	10MR
22a	Tc^6^/RL5404 Tetra RL6044	10R	5R	10R	15MR	15MR	15MR	10MR	10MR	10R	10MR
22b	Thatcher	70S	40S	60MS	80MS	80MS	70MS	70MS	80MS	50MS	45S
23	LeeFL310/Tc^6^ RL 6012	10R	10MR	20MR	30MS	50MS	30MS	40MS	50MS	50MS	40S
24	Tc^6^/Agent RL 6064	R	0	5R	R	R	R	R	R	1R	5R
25	Tc^6^/Transec RL 6084	5R	5R	20MR	20MR	20MR	20MR	20MR	30MR	20MR	40MS
26	Tc^6^/St 1.25 RL 6078	70S	40MS	60MS	80MS	70MS	70MS	70MS	80MS	70MS	60S
28	Tc^6^/C.77.1 RL 6079	10R	10R	15MR	10R	10R	10R	10R	10R	10R	40MS
29	Tc^6^/CS7D/Ag.11 RL 6080	3R	R	R	R	R	R	R	5R	5R	15S
30	Tc^6^/Terenzio RL 6049	20MR	20S	50S	80S	50S	50S	10S	20S	60S	60S
32	Tc^7^/Ae.squarrosa RL 6086	10R	10MR	20MR	20MR	20MR	20MR	10MR	10MR	10MR	15S
33	Tc^6^/PI58548(1+gene) RL 6057	60S	40S	50S	50S	50S	40S	30S	50S	30MR	25S
34	Tc^6^/PI58548 (2+gene) RL6058	70S	30S	60S	60S	50S	50S	40S	40S	30MR	20S
35	Tc^6^/RL5711	5R	5R	7R	10MR	10MR	10MR	10MR	5MR	20MR	10S
36	Neepawa^5^/T.speltoi 2-9	5R	5R	10MR	10R	R	5R	5R	5R	5R	10S
37	Tc^8^/VPM1 RL 6081	5R	5MR	10MR	10MR	10MR	5MR	5MR	5MR	20MR	35S
38	Tc^6^/TMR-S74-12-24	1R	5R	20MR	10R	10R	10R	10R	10R	10R	45S
40	KS89WGRC07RL50117	50MS	40MS	30MS	40MS	50MS	30MS	30MS	40MS	30MR	45S
39=41	TAM107*3/T.tauschi TA2460	R	R	5R	15MR	10R	10R	10R	10R	5R	5R
42	Century (Lr24)*3/T.tauschi TA2450	R	0	0	0	0	0	0	0	0	1R
43	KS92WGRC16	- *	-	-	-	-	0	0	0	0	0
43+24	TAM-200	R	0	0	R	0	0	0	0	0	1R
44	Tc^6^/T.spelta	40MR	20MR	10R	15MR	20MR	20MR	30MR	20MR	30MR	20S
45	Secale cereale RL 6144	5R	5R	5R	10R	5R	5R	5R	5R	5R	5S
47	Untitled	1R	0	R	R	R	R	R	5R	5R	0
B	Tc^6^/Carina RL6051	80S	80S	90S	80S	80S	60S	60S	60S	60S	45S
W(52)	Tc^6^/V336	5MR	5MR	10MR	20MR	20MR	10MR	10MR	10MR	10R	5R
Exch	Tc^6^/Exchange	80S	60S	60MS	80S	50S	50S	70S	60S	40MS	45S
KR1KR2	Kanred	90S	70S	80S	80MS	70MS	60MS	70MS	70MS	70S	65S
50	KS96WGRC 36	-	-	-	-	-	0	0	0	0	0
57, 58	WL711	-	-	-	-	-	-	-	10MS	10MS	15S
73	Morocco	-	-	-	-	-	-	--	0	10MS	

*—studies were not carried out due to the lack of seed material.

**Table 2:** The frequency of virulent and predominant infection types *P. triticina* isolates of the North Caucasian population to near isogenic Thatcher lines of wheat (FSCBPP greenhouse, 2011–2020).

Genes Lr	Line Names, RL	Frequency of Virulent Isolates, % (Predominant Infection Type)									
		2011	2012	2013	2014	2015	2016	2017	2018	2019	2020
1	Tc^6^/Centenario RL 6003	25.7(1)	17.6(1)	86.0(3)	40.0(2)	58.0(3)	77.6(3)	54.6(3)	86.8(3)	85.5(3)	87.8(3)
2a	Tc^6^/Webster RL 6016	49.0(2)	21.1(1)	50.0(2)	10.0(2)	30.2(1)	23.6(2)	37.3(1)	10.0(1)	23.5(2)	39.2(2)
2c	Tc^6^/Loros RL 6025	70.2(3)	47.5(2)	86.0(3)	65.0(3)	56.1(3)	74.6(3)	62.5(3)	40.0(2)	79.0(3)	52.7(3)
3	Tc^6^/Democrat RL 6002	72.3(3)	25.7(1)	99.0(3)	60.0(3)	67.7(3)	74.9(3)	66.8(3)	71.7(3)	87.4(3)	78(3)
3bg	Bage/Tc^8^ RL 6042	29.0(2)	24.9(1)	67.0(3)	20.0(2)	27.7(1)	58.5(3)	18.2(1)	55.0(2)	89.3(3)	61(3)
3ka	Tc^6^/Aniversario RL 6007	87.3(3)	40.1(1)	95.0(3)	65.0(3)	59.4(3)	75.9(3)	56.5(3)	73.3(3)	85.5(3)	86.9(3)
9	Transfer/Tc^6^ RL 6010	0(0)	0(0)	0(0)	0(0)	0(0)	0(0)	0(0)	0(0)	0(0)	12.8(0)
10	Tc^6^/Exchange RL 6004	60.0(3)	25.8(1)	29.0(2)	40.0(0)	44.3(2)	71.5(3)	38.1(2)	68.3(3)	67.4(3)	88.9(3)
11	Tc^6^/Hussar RL 6053	89.8(3)	33.5(1)	66.0(3)	30.0(2)	59.1(3)	70.1(3)	38.3(2)	70.0(3)	95.8(3)	83.8(3)
14a	Selkirk/Tc^6^	46.0(2)	27.5(1)	60.0(3)	20.0(2)	32.1(1)	56.5(3)	25.2(2)	63.3(3)	75.4(3)	91.3(3)
14b	Tc^6^/M.Escobar RL 6006	77.7(3)	40.0(1)	83.0(3)	55.0(3)	45.2(2)	64.0(3)	37.5(2)	55.0(3)	89.6(3)	64.5(3)
15	Tc^6^/Kenya W1483 RL 6052	7.7(1)	0.8(0)	21.0(1)	0(2)	4.1(1)	3.3(1)	0(0)	25.0(1)	4.8(1)	3.2(2)
16	Tc^6^/Exchange RL 6005	71.8(3)	46.7(2)	52.0(3)	35.0(2)	38.1(2)	57.5(3)	10.9(2)	68.3(3)	81.0(3)	93.8(3)
17	K. Lucero/Tc^6^ RL 6008	50.5(3)	42.9(2)	64.0(3)	20.0(2)	11.4(1)	27.0(2)	14.7(2)	75.0(3)	96.5(3)	89.1(3)
18	Tc^7^/Africa 43 RL 6090	6.7(0)	28.8(1)	66.0(3)	20.0(2)	1.5(0)	8.1(1)	11.3(0)	58.3(3)	69.5(3)	80.1(3)
19	Tc^7^/Tr.4A.elong. RL 6040	0(0)	0(0)	0.0(0)	0(0)	4.9(0)	1.3(0)	0(0)	1.6(0)	0.9(0)	9.1(0)
20	Tc^6^/Timmo RL6092	6.3(1)	6.7(0)	52.0(2)	0(0)	0(0)	24.7(1)	4.0(0)	9.5(1)	4.9(1)	47.8(2)
21	Tc^6^/RL5406 Tetra RL6043	2.0(1)	17.4(1)	22.0(2)	10.0(2)	27.0(1)	25.7(1)	14.5(0)	3.6(2)	21.8(2)	68.4(2)
23	LeeFL310/Tc^6^ RL 6012	62.8(2)	38.4(2)	43.0(2)	30.0(2)	26.0(1)	68.1(3)	35.1(2)	80.4(3)	66.8(3)	69.2(3)
24	Tc^6^/Agent RL 6064	0.5(0)	0(0)	1.0(0)	0(0)	0(0)	0(1)	0(0)	20.0(1)	0(1)	14.8(1)
25	Tc^6^/Transec RL 6084	5.8(1)	7.7(1)	9.0(1)	15.0(1)	0(1)	22.9(1)	10.3(1)	60.8(3)	35.8(2)	81.0(3)
26	Tc^6^/St 1.25 RL 6078	58.3(3)	43.8(2)	60.0(3)	30.0(2)	22.8(2)	55.1(3)	27.8(1)	73.3(3)	72.6(3)	84.2(3)
28	Tc^6^/C.77.1 RL 6079	2.0(1)	36.7(1)	55.0(3)	45.0(2)	36.7(1)	39.7(2)	23.6(1)	55.0(3)	24.4(1)	83.2(3)
29	Tc^6^/CS7D/Ag.11 RL 6080	0(1)	0.8(0)	2.0(0)	0(0)	21.4(0)	0(0)	2.6(0)	0(1)	2.3(1)	10.0(1)
30	Tc^6^/Terenzio RL 6049	78.7(3)	63.2(3)	69.0(3)	60.0(3)	1.5(2)	75.4(3)	45.8(2)	73.3(3)	40.1(2)	91.4(3)
32	Tc^7^/Ae.squarrosa RL 6086	1.8(1)	1.6(1)	5.0(1)	0(1)	44.9(1)	0(1)	22.9(1)	22.1(2)	12.3(2)	26.2(2)
33	Tc^6^/PI58548(1+gene) RL 6057	66.7(3)	46.0(2)	65.0(3)	50.0(3)	13.5(2)	61.2(3)	38.7(2)	62.6(3)	58.8(3)	92(3)
36	Neepawa^5^/T.speltoi 2-9	35.5(2)	29.4(2)	17.0(2)	5.0(1)	64.2(3)	33.9(2)	7.6(2)	78.7(3)	54.8(3)	74.8(3)
38	Tc^6^/TMR-S74-12-24	1.0(0)	12.5(1)	71.0(3)	0(0)	34.9(0)	1.2(0)	3.9(0)	77.3(3)	31.3(1)	9.2(1)
40	KS89WGRC07RL50117	70.5(3)	47.2(2)	79.0(3)	45.0(2)	2.0(1)	60.4(3)	54.7(3)	68.5(3)	46.3(2)	96.2(3)
41	TAM107*3/T.tauschi TA2460	0(0)	0(0)	0(0)	0(0)	48.1(1)	8.8(0)	2.6(0)	2.7(0)	0(1)	2.2(1)
42	Century (Lr24)*3/T.tauschi TA2450	0(0)	0(0)	0(0)	0(0)	0(0)	0(0)	0(0)	7.5(1)	1.7(1)	2.4(1)
43+24	TAM-200	0(0)	0.7(0)	0(0)	0(0)	0(0)	1.0(0)	6.4(0)	4.7(0)	9.1(0)	0(0)
44	Tc^6^/T.spelta	3.2(1)	5.6(1)	12.0(2)	5.0(2)	4.7(1)	42.0(2)	44.8(1)	36.7(2)	31.4(2)	43.7(2)
45	Secale cereale RL 6144	1.8(1)	0(1)	0(1)	10.0(1)	33.7(0)	11.5(1)	14.1(1)	11.0(1)	16.3(2)	47.7(2)
47	Untitled	0(0)	0(0)	0(0)	0(0)	0(0)	0(0)	0(0)	21.5(1)	5.1(1)	3.0(1)
B	Tc^6^/Carina RL6051	72.5(3)	69.4(3)	84.0(3)	55.0(3)	53.1(3)	72.6(3)	45.3(2)	81.8(3)	64.6(3)	91.3(3)
W(52)	Tc^6^/V336	0.7(1)	2.0(1)	1.0(1)	0(1)	8.4(1)	2.9(1)	2.5(1)	6.8(1)	9.6(1)	33.5(2)
Exch	Tc^6^/Exchange	88.0(3)	49.1(2)	68.0(3)	55.0(3)	53.2(3)	56.6(3)	53.5(3)	59.5(3)	60.8(3)	59.3(3)
KR1KR2	Kanred	96.0(3)	89.0(3)	93.0(3)	60.0(3)	62.4(3)	69.1(3)	56.2(3)	17.3(2)	62.8(3)	85.2(3)
50	KS96WGRC 36	-	-	-	0(0)	0(0)	0(0)	0(0)	0(0)	13.4(1)	41.5(2)

## Data Availability

All data obtained is contained in this article.
